# Where is my sample? Investigating pre-analytical pathology sampling errors in a psychiatric hospital

**DOI:** 10.1192/bjo.2021.911

**Published:** 2021-06-18

**Authors:** Emma Scholes-Pearson, Nieves Mercadillo

**Affiliations:** 1 Warrington and Halton Teaching hospitals; 2 North West Boroughs Healthcare NHS Foundation Trust

## Abstract

**Aims:**

Errors in the pathology sampling process can be costly for all stakeholders in any clinical setting; however, this process is often overlooked within psychiatry. Over the space of just a few short months at Hollins Park Psychiatric Hospital (HPH) such errors were reported to be numerous with staff raising multiple issues relating to the pathology sampling process. These issues often had a negative impact on patient care, leading to outcomes such as as re-bleeding of patients and delays in interventions. Here, we aim to identify the predominant sources of error in this process and suggest possible improvements to minimise these errors in the future.

**Method:**

Initially, we mapped and analysed each step of the sampling process as it is currently performed in order to identify areas of possible improvement. We then distributed questionnaires to all junior doctors - who are responsible for the handling of samples within the clinical setting – in order to establish error type and frequency. Questions also assessed individual confidence and familiarity with the sampling process.

**Result:**

When mapping the sampling process, we identified all key steps required when sending samples from HPH to Warrington and Halton Hospitals laboratory. This included one pathway for sending routine bloods, and one pathway for urgent bloods. The process for sending routine bloods required more steps and ultimately took longer for samples to reach the laboratory – as expected. Of the issues identified during mapping of the pre-analytical phase, a majority of 77.7% of clinicians reported samples had gone missing or were unreported – with the reasons for this being undetermined in most cases – and 55.5% reported their samples never reached the lab. While on the whole participants were comfortable with the steps involved in sending samples to the laboratory, 77.7% were not aware of the requirement to log samples as they were being sent.

**Conclusion:**

The reasons underlying errors in the sampling process at HPH were multifactorial and included a lack of clinician familiarity with correct procedure, poor sample recording/tracking and lengthy transit times between the patient and laboratory. Here we outline some simple evidence-based recommendations (including education of staff and improved tracking through an electronic requesting system) to help reduce errors and streamline the sampling process in the hopes of improving both efficiency and accuracy, reducing the financial and clinical impact.

